# A framework for feature extraction from hospital medical data with applications in risk prediction

**DOI:** 10.1186/s12859-014-0425-8

**Published:** 2014-12-30

**Authors:** Truyen Tran, Wei Luo, Dinh Phung, Sunil Gupta, Santu Rana, Richard Lee Kennedy, Ann Larkins, Svetha Venkatesh

**Affiliations:** Centre for Pattern Recognition and Data Analytics, Deakin University, Geelong, VIC 3220 Australia; Department of Computing, Curtin University, Perth, WA Australia; School of Medicine, Deakin University, Geelong, VIC Australia; Barwon Health, Geelong, VIC Australia

**Keywords:** Feature extraction, Risk prediction, Hospital data

## Abstract

**Background:**

Feature engineering is a time consuming component of predictive modeling. We propose a versatile platform to automatically extract features for risk prediction, based on a pre-defined and extensible entity schema. The extraction is independent of disease type or risk prediction task. We contrast auto-extracted features to baselines generated from the Elixhauser comorbidities.

**Results:**

Hospital medical records was transformed to event sequences, to which filters were applied to extract feature sets capturing diversity in temporal scales and data types. The features were evaluated on a readmission prediction task, comparing with baseline feature sets generated from the Elixhauser comorbidities. The prediction model was through logistic regression with elastic net regularization. Predictions horizons of 1, 2, 3, 6, 12 months were considered for four diverse diseases: diabetes, COPD, mental disorders and pneumonia, with derivation and validation cohorts defined on non-overlapping data-collection periods.

For unplanned readmissions, auto-extracted feature set using socio-demographic information and medical records, outperformed baselines derived from the socio-demographic information and Elixhauser comorbidities, over 20 settings (5 prediction horizons over 4 diseases). In particular over 30-day prediction, the AUCs are: COPD—baseline: 0.60 (95% CI: 0.57, 0.63), auto-extracted: 0.67 (0.64, 0.70); diabetes—baseline: 0.60 (0.58, 0.63), auto-extracted: 0.67 (0.64, 0.69); mental disorders—baseline: 0.57 (0.54, 0.60), auto-extracted: 0.69 (0.64,0.70); pneumonia—baseline: 0.61 (0.59, 0.63), auto-extracted: 0.70 (0.67, 0.72).

**Conclusions:**

The advantages of auto-extracted standard features from complex medical records, in a disease and task agnostic manner were demonstrated. Auto-extracted features have good predictive power over multiple time horizons. Such feature sets have potential to form the foundation of complex automated analytic tasks.

**Electronic supplementary material:**

The online version of this article (doi:10.1186/s12859-014-0425-8) contains supplementary material, which is available to authorized users.

## Background

In their latest book that has attracted wide attention [1], Mayer-Schonberger and Cukier argued that we are transitioning from a hypothesis-driven small-data world—where data are purposely collected to validate a hypothesis—to a data-driven big-data world—where more scientific discoveries will be driven by the abundance of data collected for other purposes. The same trend is observed in healthcare and biomedical research. Although randomized control trials with primary data collection will continue to be the gold standard, hypothesis generation and quality improvement based on the routinely collected patient records have demonstrated great potentials [2-4].

Despite increasing awareness of their potential values in risk modeling, hospital data are still, to a large degree, under-exploited [5,6]. A major obstacle lies in the diversity and complexity of patient records. Different medical specialties will collect disease-specific data—for example, suicide risk assessments have a different data format from white-blood-cell counts. Hand picking features (independent variables) for each analysis is clearly not efficient, and it also cannot guarantee that all important information in the existing data is included.

In recent years, various risk indices or scores have been developed, competing to become the feature set for risk modeling [7]. These include comorbidity based Elixhauser index [8], Charlson index [9] and variants [10,11], and physiology-driven Tabak score [12]. However, as these indices were developed with a small number of target diseases, it is unknown how they can be generalized to other diseases. In literature, different features were still chosen in different studies [13], which make it difficult to compare among different models.

As pointed out by Cukier [14], one important advantage of big data is the ability to look into thousands of factors at the same time, even those seemingly “extrinsic” to the problem at hand (e.g., Vioxx, an arthritis pain reliever, to heart attack before the Kaiser Permanente study [15]). In other words, reducing a patient’s medical history into a small number of known “intrinsic” risk factors may be unnecessary, in some cases even wasting important information. With emerging machine learning techniques that can handle a huge number of independent variables (known as the *p* ≫ *n* problem [16]), it suffices to maximally include information related to patient history in the database. But can such encoding of diverse patient information be compiled with minimal human involvement and be updated when new data are collected?

In this article, we propose a disciplined framework that converts diverse patient information in an administrative database into a set of inputs suitable for machine-learning risk modeling. The core idea is to treat a patient’s medical history as a bank of signals recorded since the first patient encounter, with each signal corresponding to a particular clinical event. The signal concept provides a unifying format on which a set of generic filters can be applied to extract feature vectors. This is in contrast with what commonly understood as feature generation (e.g., [17]), where 1) data are often short-term and with a well-defined format (e.g., physiological signals), and 2) knowledge of specific medical conditions is often essential (e.g., [18]). In our framework, the definitions of the signals were specified through an entity schema for the whole administrative database—this is the only manual step in the framework. The separation of the schema construction step avoids repeated manual work for each medical condition, and allows organic grow of the feature sets and easy incorporation of domain knowledge.

To evaluate the feature extraction framework, we considered the application of readmission prediction. Readmission is common following hospitalization for common medical conditions [19,20]. There is a recognized need for cost-effective, targeted interventions to decrease avoidable readmissions [21]. A means of identifying patients at high-risk of readmission would be of great benefit, but prediction tools described to date have proved to perform relatively poorly [13].

## Methods

### Ethics statement

Ethics approval was obtained from the Hospital and Research Ethics Committee at Barwon Health, with whom Deakin University has reciprocal ethics authorization (approval number 12/83). Written consent was obtained from the patients for their information to be stored in the hospital database and used for research.

### Temporal feature extraction: framework description

For various pieces of patient information, a simple dichotomy will affect how they are used in a big-data algorithm. That is, a piece of patient information is either constant (in the course of illness) or temporally varying. The former group includes patient’s gender and other demographical information; the latter group includes time-stamped events such as hospitalizations, emergency visits, and abnormal lab tests. Static information can be easily incorporated into a flat-data format that most statistical models expect. In contrast, temporal information is more difficult to handle. Time-series models (e.g., [22]) or smoothing methods (e.g., [23]) work in simple analyses with a handful of variables, but break when used in a big-data setting with thousands of both discrete and continuous features.

**Figure 1:**
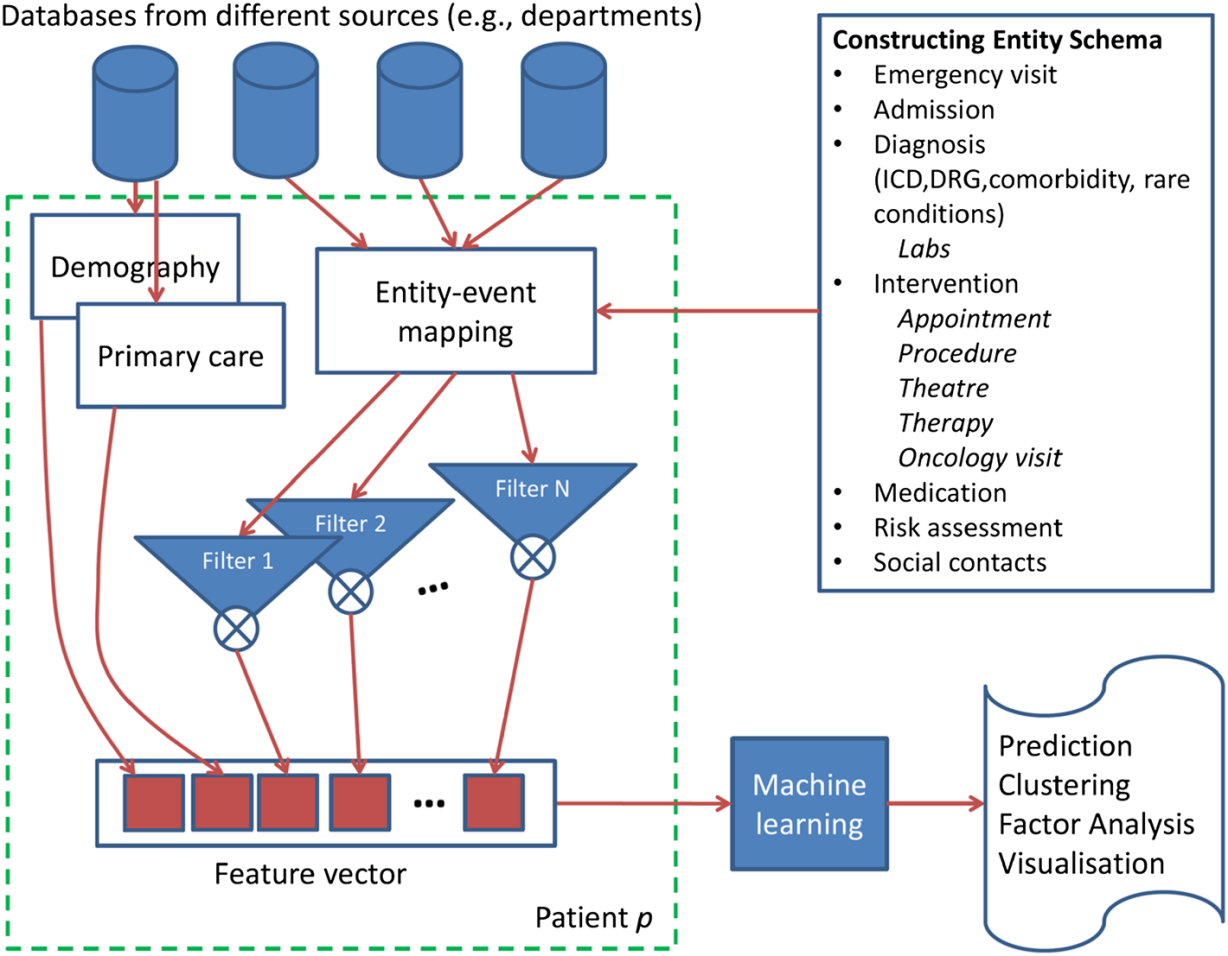
**Overview of the feature extraction framework.**

The temporal feature extraction consists of three steps: constructing an entity schema, generating event time series, and applying convolution filters (See Figure 1).

### Construction of the entity schema

At the center of the framework we propose is the observation that most temporal information is stored in an administrative database as time-stamped database entities. An emergency visit, a hospitalization, or an abnormal blood test all has a time stamp. Therefore, a patient history can be reconstructed by scanning an administrative database for time-stamped entities, which results in a set of event sequences, one for each entity type.

**Figure 2:**
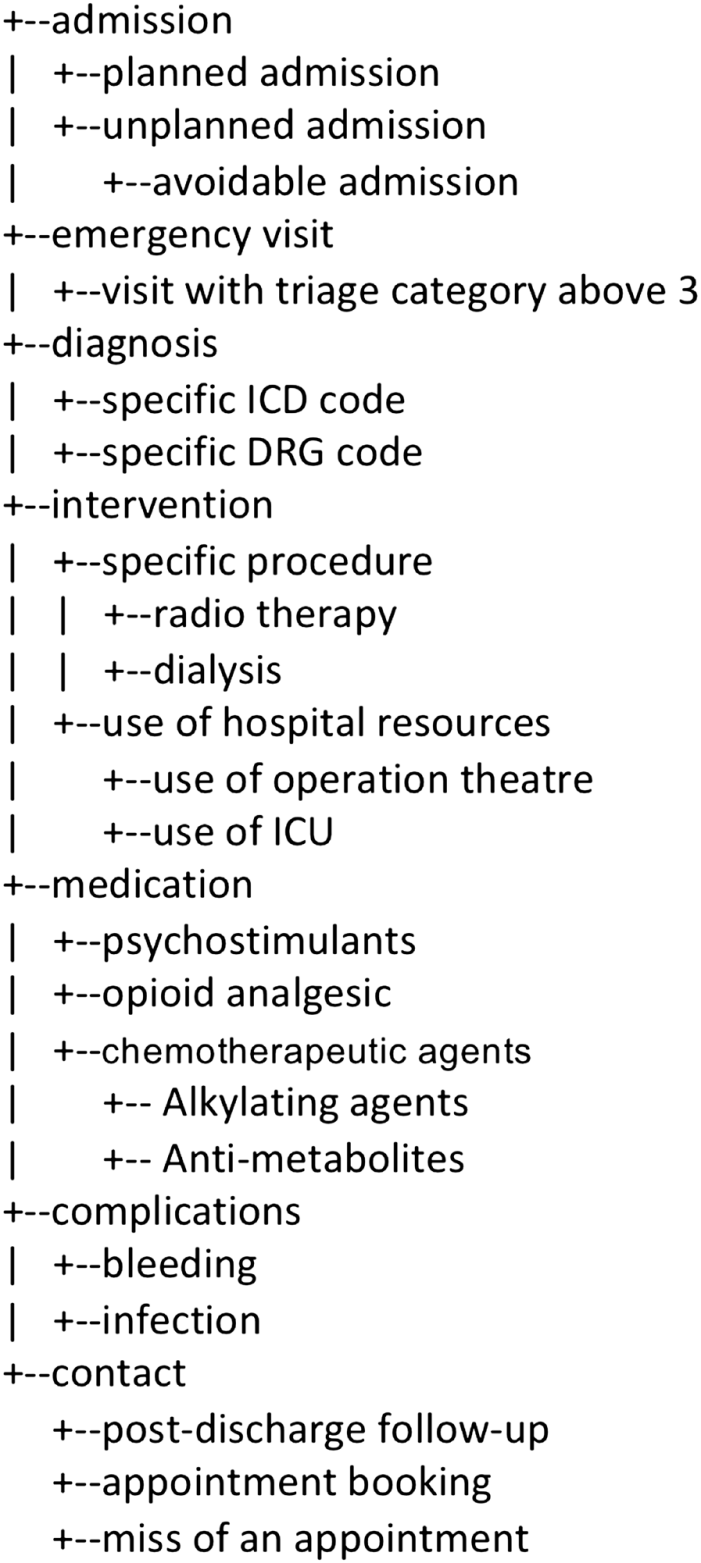
**An example subsection of an entity schema.** Not all entities were used by the experiments in this paper.

For each administrative database, entities of interest can be identified and grouped into functionally similar groups, resulting in an entity schema (see Figure 2 for an illustration). In practice, the schema can be built iteratively. One can start with a basic schema derived from existing meta-data (e.g., an existing medical ontology) and keep adding more entities when richer features are needed. For example, the schema can include diagnoses encoded using International Statistical Classification of Diseases (ICD [24]), procedures encoded using Australian Classification of Health Interventions [25], medications encoded using the Anatomical Therapeutic Chemical classification [26], comorbidities encoded using the Elixhauser comorbidity index [8], admissions encoded using diagnosis-related groups [27]. In defining the schema, the mapping from data to entities can be realized using SQL snippets. As the result of the mapping, each instance of an entity will have a number of attributes, including the source record id (e.g., patient UR) and the timestamp for the corresponding event.

Because the function of the schema is to define features for a prediction task, redundant information is allowed and should actually be encouraged. The modern predictive modeling techniques such as random forests can handle a large number of redundant independent variables, without running into the collinearity problem faced by traditional regression models. Because of this tolerance to redundant entity definitions, the schema can be jointly constructed and improved by multiple users—for example both IT staff and clinical nurses with inputs from medical specialists.

Note that contents in such an entity schema are primarily driven by the database, not by the risk modeling tasks. Hence the resulted schema can be used for different risk models. This data-driven approach is in contrast with application-driven feature extraction in the small-data world, where a small number of data columns are first selected according to their relevance to the particular analytical task at hand.

### Generation of personalized event time series

Once the entity schema is constructed, the database is scanned to look for entities and associated time stamps in the medical history of a person, in an automated fashion. We distinguish two types of temporal entities: point entities (e.g., first diagnosis, resection of tumors) and continuing entities which cover a duration (e.g., an episode of hospital stay). A pair (time, occurrence of entity) is defined as an *event* of that entity. The time dimension is first discretized using a minimum time unit, thus a continuing entity may correspond to multiple events through temporal discretization.

Discretization is also driven the need for efficient processing. For modeling of readmission risk, for example, dicretization by days is a convenient option that often suffices. Then the time dimension becomes a sequence 1, 2, … , *T*, where *T* is the maximum length of patient history of interest. Given an entity *i*, a time series *E*_*i*_ is constructed such that each value *E*_*i*_*(t)* counts the number of occurrences of the entity during the time interval *t*.

**Figure 3:**
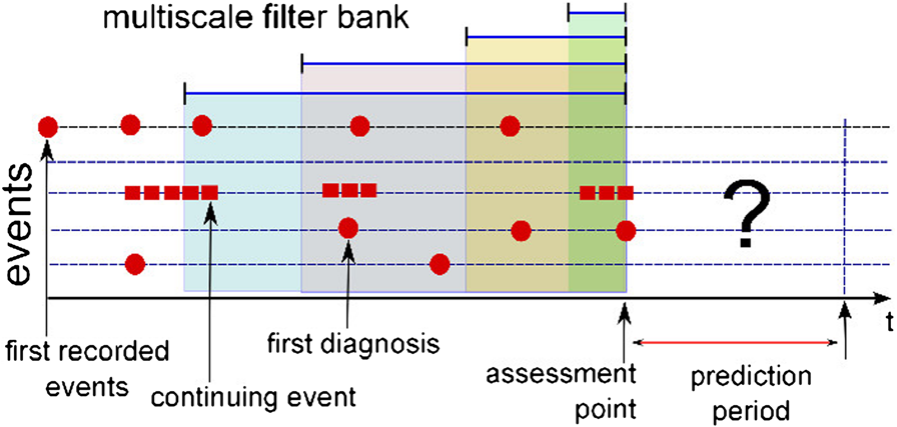
**Temporal data represent events in a patient history.** Assessment point (AP) defines the timestamp from which future readmissions within a pre-defined period are predicted. Events occurring before the AP were used to construct features. Only APs after the first diagnosis of the disease under study were considered.

Therefore each entity in the schema corresponds to an event sequence (See Figure 3). This common representation of diverse temporal events forms the basis of the automated feature extraction to be presented in this article. This is akin to an “image” of clinical events.

### Aggregation with multi-resolution one-side filter bank

As different events may affect patient risk at different paces, a set of filters of different bandwidths are applied to smooth an event time series. In the engineering discipline of signal processing, such multi-resolution analysis is an effective way to extract multiple underlying signal components of varying time scales (also known as frequencies, see for example [28]). In our framework, such filters provide a principled way to aggregate events in time periods of interest, and at the same time, uncovering the smooth progression of diseases from multiple discrete event sequences. Different from the traditional filter design, our filters are *one-sided*, i.e., only historical events are considered.

A one-sided filter bank is a set of *convolution kernels* {*k*_*i*_, 1 ≤ *i* ≤ *N*} with different bandwidths *σ*_*i*_. They are functions of (near) zeros everywhere except for a small interval defined by *σ*_*i*_ after the origin. For a time series of event counts, kernels are used to achieve aggregation and denoising. The simplest kernel is the uniform function (also known as rectangular windows):

$$ {K}_i(t)=\left\{\begin{array}{l}\frac{1}{\sigma_i},\kern1em 0\le t\le {\sigma}_i\hfill \\ {}0,\kern1.48em \mathrm{otherwise}.\hfill \end{array}\right. $$

Alternatively, a one-sided Gaussian kernel can be used to highlight the effect of recent events:

$$ {K}_i(t)=\left\{\begin{array}{l}\sqrt{\frac{2}{\pi {\sigma}_{{}_i}^2}{e}^{-\frac{t^2}{2{\sigma}_{{}_i}^2}},\kern1em t>0}\hfill \\ {}0,\kern2.32em \mathrm{otherwise}.\hfill \end{array}\right. $$

For each evaluation point *t*, a set of features are extracted, one feature for each kernel:

$$ {X}_i(t)={\displaystyle \sum_{h=0}^{t-1}{K}_i\left(h-{h}_i\right) \cdot p\;{E}_i\left(t-h\right),} $$

where 1 ≤ *i* ≤ *N* and *h*_*i*_ ≥ 0 denotes delay. The delay operator is equivalent to shifting the kernel backward. The effect is that only the history before the point *t* − *h*_*i*_ is accounted for. By varying *h*_*i*_, the temporal progression of entities (e.g., diseases) is captured.

In the end, a total of *M* × *N* temporal features are extracted per evaluation point, where *M* denotes the number of event time series and *N* denotes the size of the filter bank. In this study, uniform kernel was used as it gives intuitive interpretation of time interval in which a particular event occurs. The pairs (bandwidth *σ*_*i*_, delay *h*_*i*_), in months, were: (1, 0), (3, 0), (6, 0), (12, 0), (12, 12), (12, 24). That is, a filter bank of *N* = 6 elements and the history of 36 months before each evaluation point were considered.

The number of event sequences *M* was controlled by using two heuristics. First, rare events of the same type were not considered separately but were grouped into an extra event. In our setting, an event was considered rare if it occurred less than 50 times in the database. Second, a maximum dictionary size was imposed for each event type, that is, only the top 2,000 most popular events of each type were considered. The rest was treated was rare events.

Features extracted through this method are termed *auto-extracted features*, reflecting that all feature-extraction steps after the initial schema creation are automated.

### Overall system

The overall system is shown in Figure 1. For each patient, datasets from different sources are input. The entity-to-event mapping followed by convolution with a filter bank produces a rich set of features that can be input to a machine learning system for diverse tasks - predicting readmissions, understanding factors, cohort clustering or visualization.

### Comparison with Elixhauser-comorbidities-based features: framework evaluation

In the evaluation, a feature set was extracted from administrative data for a mega cohort consisting of four patient sub-cohorts: COPD (Chronic Obstructive Pulmonary Disease), Diabetes, Mental Disorder, and Pneumonia. The four cohorts were chosen due to their perceived burden in hospital readmission. For each sub-cohort, the feature set was applied to model the risk of readmission. For comparison, purposely-designed features (risk factors) for each cohort were also applied in parallel. For the feature extraction framework to have practical values, the automatically extracted features need to demonstrate at least the same level of discriminative power with those handcrafted.

Data were extracted from electronic records of inpatient admissions and emergency department (ED) visits at Barwon Health, a regional health service in Australia. As the only tertiary hospital in Greater Geelong, a catchment area with more than 350,000 residents, the hospital’s patient database provides a single point of access for information on patient hospitalizations, ED visits, and in-hospital medications. In hospital medications were aggregated in several levels, using the Anatomical Therapeutic Chemical (ATC) hierarchy [26]. The four patient sub-cohorts are defined as follows:

1. **Diabetes** (ICD-10 code block: E10-E14)
2. **COPD** (ICD-10 code block: J44)
3. **Mental Disorders** (ICD-10 code block F00-F99)
4. **Pneumonia** (ICD-10 code block J12-J18)

The risk-modeling task is the prediction of unplanned readmissions after an assessment point (AP) within various prediction horizons (see Figure 3). Here unplanned readmission was defined to be readmissions following an emergency visit. (It is challenging to ascertain the exact nature of a patient admission using hospital data. Our definition is mostly driven by pragmatism, but we feel that it serves the purpose of evaluating our feature construction framework.) The AP can be the discharge date for a particular admission or a pre-defined date. Only APs after the first diagnosis of the given disease were considered. Thus, each AP defines a unit of analysis: data prior to the AP were used to derive the feature sets (independent variables); readmissions following the AP define the dependent variable.

APs from each cohort were split into a derivation set and a validation set. To achieve the best estimate of performance generalization, the derivation and the validation sets were separated *both* in patients and in time. First, the patient’s events were divided by the validation point. Patients whose APs occurred before the validation point formed the derivation cohort. Their subsequent APs after the validation point were not considered. The other patients formed the validation cohort.

The Elixhauser comorbidities [8] were used as a baseline feature set. These 30 comorbidities were chosen for their relevance in multiple patient groups and can be mapped from ICD-10 codes using the algorithm in [29]. A recent meta-analysis [7] shows that the Elixhauser comorbidities have higher discriminative power compared with competing comorbidities. With that said, the Elixhauser comorbidities depend solely on ICD codes; other information in administrative data, such as medications, is not captured. In mapping ICD codes to the Elixhauser comorbidities, the codes from all hospitalizations within a period before the APs are considered. This compensates for the likelihood of miscoding certain conditions in administrative data. Two periods prior to the prediction points were considered: 1 month and 3 years; they correspond to two baseline feature sets in Table 1.

**Table 1:** **Feature sets, baseline and auto-extracted**

**Feature set**	**Data Used**
**Baseline (1 M)**	Elixhauser comorbidities + demography, present over 1 month history
**Baseline (3Y)**	Elixhauser comorbidities + demography, present over 36 months history
**Auto-extracted Set MR**	MR + demography, over 36 months history
**Auto-extracted Set MR+ Comorbidities**	MR + demography + Elixhauser comorbidities, 36 months history

From the database, following the entity-event mapping, two feature sets were constructed: Set MR (for Medical Records) and Set MR + Comorbidities. Set MR was derived from all available historical clinical data including GPs, insurances, admissions, emergency visits, ICD, DRG, procedure, medication, surgery codes and higher level categories for them (see Figure 2 for more comprehensive list). The second set (MR + Comorbidities) was the Set MR augmented with the Elixhauser comorbidities. Both feature sets were complemented with basic socio-demographic features, such as age, gender, and postcodes (see Figure 1 for the construction of the combined features from different sources).

To compare the amount of information captured in the Elixhauser comorbidities and the automatically extracted feature sets, a common prediction method was used on all feature sets. The method consists of logistic regression with elastic net regularization [30], which is a widely accepted method for handling thousands of variables and producing robust prediction. Features were normalized to the unit interval [0,1], and then transformed using square root. The primary performance measure was AUC (Area Under the ROC Curve, also equivalent to the *c*-statistic) and its Mann-Whitney’s 95% confidence intervals.

Four features sets, 2 baseline and 2 auto-extracted feature sets, are summarized in Table 1.

## Results

8,445 features were automatically extracted from the dataset. The feature extraction step took less than five minutes on a standard desktop PC with 4 cores and 8 GB of memory (The database was a SQL Server 2005 instance and the algorithm was implemented in Perl). Results reported in the main text are for the assessment points at the discharges following the first diagnosis of the diseases under study (Diabetes, COPD, Mental Disorders and Pneumonia). Results for assessments points at pre-defined times, as well as the discovered features, are presented in the Additional file 1.

The numbers of analysis units for different cohorts were summarized in Table 2. Basic characteristics of the cohorts are summarized in Table 3.

**Table 2:** **Definition of derivation and validation cohorts and the distribution of analysis units in the cohorts (evaluated at discharges following the first diagnosis)**

	**Derivation cohort**	**Validation cohort**
**Diabetes**		
Period	2003-2007	2008-2011
Number of patients	4,930	2,101
Number of analysis units	11,897	4,041
**COPD**		
Period	2003-2008	2009-2011
Number of patients	1,816	1,816
Number of analysis units	5,746	5,270
**Mental disorders**		
Period	2003-2009	2010-2011
Number of patients	3,089	1,248
Number of analysis units	10,728	2,232
**Pneumonia**		
Period	2003-2008	2009-2011
Number of patients	3,258	2,264
Number of analysis units	7,817	4,020

**Table 3:** **Characteristics in patient cohorts**

**Medical Condition**	**Derivation cohort**	**Validation cohort**
**Diabetes**		
Average Age	67.6	66.1
Gender Distribution (% of females)	45.3	43.1
Median time to readmission (months)	5.7	8.4
**COPD**		
Average Age	74.9	72.0
Gender Distribution (% of females)	41.8	42.4
Median time to readmission (months)	4.1	4.8
**Mental disorders**		
Average Age	48.9	49.9
Gender Distribution (% of females)	50.8	48.6
Median time to readmission (months)	4.9	6.4
**Pneumonia**		
Average Age	67.0	63.9
Gender Distribution (% of females)	44.8	46.2
Median time to readmission (months)	5.6	8.9

Table 4 shows results using the four feature sets on readmission in four diverse cohorts. Readmission horizons of 1, 2, 3, 6, and 12 months are considered.

**Table 4:** **Performance (AUC) of predicting unplanned readmissions following the unplanned discharges**

		**BASELINE (95% CI)**			
	**Period**	**1 M**	**3Y**	**MR (95% CI)**	**MR + Comorbidities (95% CI)**
**COPD**					
	1 M	0.57 (0.55,0.60)	0.60 (0.57,0.63)	0.730 (0.695,0.766)	0.730 (0.695,0.766)
	2 M	0.59 (0.56,0.61)	0.60 (0.57,0.62)	0.719 (0.689,0.750)	0.719 (0.689,0.750)
	3 M	0.58 (0.56,0.61)	0.60 (0.58,0.63)	0.719 (0.692,0.746)	0.720 (0.693,0.746)
	6 M	0.59 (0.57,0.61)	0.61 (0.59,0.64)	0.724 (0.703,0.746)	0.724 (0.702,0.745)
	12 M	0.60 (0.57,0.62)	0.62 (0.59,0.64)	0.720 (0.701,0.739)	0.720 (0.701,0.739)
**Diabetes**					
	1 M	0.60 (0.57,0.62)	0.60 (0.58,0.63)	0.708 (0.674,0.741)	0.704 (0.670,0.738)
	2 M	0.61 (0.59,0.63)	0.63 (0.61,0.65)	0.718 (0.692,0.744)	0.718 (0.692,0.743)
	3 M	0.60 (0.58,0.622)	0.63 (0.61,0.65)	0.724 (0.703,0.745)	0.724 (0.703,0.745)
	6 M	0.62 (0.60,0.633)	0.64 (0.62,0.66)	0.714 (0.697,0.731)	0.715 (0.698,0.732)
	12 M	0.64 (0.62,0.653)	0.66 (0.64,0.68)	0.718 (0.705,0.732)	0.718 (0.704,0.732)
**Mental disorders**					
	1 M	0.56 (0.53,0.59)	0.57 (0.54,0.60)	0.748 (0.709,0.787)	0.747 (0.708,0.786)
	2 M	0.58 (0.55,0.61)	0.60 (0.57,0.62)	0.756 (0.727,0.784)	0.756 (0.728,0.785)
	3 M	0.59 (0.57,0.62)	0.60 (0.58,0.63)	0.738 (0.713,0.764)	0.737 (0.711,0.762)
	6 M	0.61 (0.59,0.64)	0.63 (0.61,0.65)	0.718 (0.697,0.740)	0.718 (0.696,0.739)
	12 M	0.65 (0.63,0.67)	0.66 (0.64,0.68)	0.713 (0.694,0.732)	0.713 (0.694,0.732)
**Pneumonia**					
	1 M	0.58 (0.55,0.60)	0.61 (0.59,0.63)	0.749 (0.717,0.782)	0.750 (0.718,0.782)
	2 M	0.61 (0.59,0.63)	0.66 (0.64,0.68)	0.753 (0.729,0.777)	0.756 (0.733,0.780)
	3 M	0.62 (0.60,0.64)	0.67 (0.65,0.68)	0.760 (0.739,0.780)	0.762 (0.742,0.782)
	6 M	0.64 (0.62,0.66)	0.68 (0.67,0.70)	0.748 (0.731,0.764)	0.749 (0.733,0.765)
	12 M	0.65 (0.63,0.67)	0.70 (0.68,0.71)	0.744 (0.731,0.758)	0.747 (0.733,0.761)

AUC stands for Area Under ROC Curve; Feature sets are Elixhauser comorbidities as baselines, automatically extracted features from medical records (MR), and the combination of MR and comorbidities.

On all cohorts, the automatically extracted features resulted in better prediction accuracy, measured in AUC, indicating better capturing of the information in discharge diagnoses.

## Discussion

Our results confirm that auto-extracted disease-agnostic features from hospital medical data can achieve better discriminative power than carefully crafted comorbidity lists.

By generating features from administrative databases, many data mining and machine learning algorithms that expect flat-table features can now be applied for a broad range of risk modeling tasks—from readmission prediction to cancer survival prognosis. As data preparation often accounts for 60%-90% of time in data analysis [31], our framework has potential in greatly reducing analytics cost in health care.

The number of features generated is constrained by the size of the entity schema. As the entity schema has a tree structure, the number of entities in the schema is at most twice the number of the most granular entities. With a specific application, these features generated from the generic entities can be further processed if domain knowledge exists for guiding “pattern” generation. For example, knowledge about clinical domains was applied to drive temporal feature extraction [18]. (However such domain knowledge is not always readily available for a hypothesis-generating data-mining task.)

A slightly surprising result was that adding Elixhauser comorbidities into the auto-extracted feature set did not increased predictive power. This may be due to that the Elixhauser comorbidities were themselves mapped from ICD-10 codes, and hence contained no additional information.

The feature extraction step precedes the analysis task and is disease agnostic. Therefore the auto-extracted features make it easier to model risk *across different medical conditions*—analyses with pooled data have become more common [32-34]. This advantage is demonstrated by our results on the aggregated mental disorder cohort. This disease-agnostic property is particularly valuable for diseases that are not yet sufficiently studied.

It is difficult to validate the best readmission prediction performance reported in the literature. In the previously mentioned systematic review of 26 models [13], only two models from [35] had achieved an AUC (c statistics) higher than 0.72, but on predicting “complicated care transitions” as defined by the authors, which are different from readmissions. In addition, we divided the derivation cohort and the validation cohort by time, in contrast to random partition in most previous studies. Although models can be updated weekly or even more frequently, the update cannot capture future trends of the readmission rates, especially when we are predicting readmission in a longer term—for example—the next 3 years. The uncertainty of future readmission rates, we believe, should be accounted for by using a temporally non-overlapping validation set. This choice may lead to lower reported prediction accuracy, but it helps avoid unwarranted confidence towards our prediction model. Actually in Australia, the time-to-readmission has been steadily increasing (and consequently the readmission rate has been decreasing) over the years due to service improvements (see Table 3), the prediction task we set for ourselves is more realistic, but also more challenging. In this sense, our automatically extracted features have predictive power better than the best feature sets crafted to predict readmission.

In many fields, significant resources has been invested to develop standardized feature sets, as people believed that a small number of features form a necessary bridge between raw data and knowledge. In image processing, such standardized feature sets, called *low level features* (e.g., SIFT [36]), are critical in the complex task of image understanding and provide a *task* independent way to deal with the diversity, complexity and volume of data. In the big data world, limiting the number of features is no longer possible and no longer necessary, thanks to the emerging machine learning algorithms for extremely high dimension data. We believe that our automated feature sets are the equivalent of *low level features* in administrative data, but the size of the feature set can grow with the data. They provide a principled way to extract features in a disease and task invariant way, laying a crucial foundation for more complex tasks.

The clinical implications of our framework lie in the renewed possibility of highly accurate prediction of individual risk, through exploiting all possible raw data available for a particular patient, especially when he or she has uncommon medical conditions. We have applied the techniques reported here in predicting suicidal behaviors, and the initial results were very encouraging. We are also in the process of applying auto-extracted features to help a health service tailor risk profiles of Diabetes/COPD patients, potentially resulting in better utilization of costly medical resources.

One contribution of our framework in the big-data context is the notion of entity schema for time-stamped data. This enables parallel scanning of a database for temporal events. Defining each event is a key-value pair of a time stamp and an entity allows a MapReduce scheme to be applied for scalability.

For the simplicity of evaluation, only readmission modeling was considered. Another common risk modeling task is for mortality prediction. As mortality information is not routinely collected in administrative databases, evaluation is more difficult. However, with a broadly defined entity schema, the same feature set for readmission prediction can also be applied to mortality prediction.

Risk estimation remains central to medical care. A paradigm shift, to consider thousands of factors is now open through electronic medical records. We proposed a framework for automated feature extraction, in a task and disease independent manner.

## Conclusions

We proposed a framework that generates features for machine-learning risk modeling from administrative databases. The framework does not rely on a pre-existing data warehouse. It allows organic growth of the feature sets and easy incorporation of domain knowledge. The process is efficient and task-agnostic. The auto-extracted disease-agnostic features from the hospital databases achieve better discriminative power than carefully crafted comorbidity lists.

## Additional file

Additional file 1:
**Additional results: Prediction performance and top features.**
