# Supplementary material for: A framework for feature extraction from hospital medical data with applications in risk prediction
Source: BMC Bioinformatics. 2014 Dec 30;15(1):425. doi: 10.1186/s12859-014-0425-8 (PMC4310185; doi:10.1186/s12859-014-0425-8)
Supplement: Additional file 1: — Additional results: Prediction performance and top features. [file 12859_2014_425_MOESM1_ESM.doc]

# Additional file

## Prediction performance with different settings.

We evaluated the auto-generated features on the task of predicting unplanned readmissions in two different settings. In the first setting, each discharge was a unit of analysis (points of prediction); In the second setting, the first day of each calendar year was a unit of analysis. The manuscript reported the results for the first setting; the results for the other setting are reported as follows.

Table 1 Cohort definitions for the second setting: prediction on the first day of years following the first diagnosis.

|  | **ICD-10 code** | **Train/test cohorts** | **Train/test prediction points** | **Train/test period** |
| --- | --- | --- | --- | --- |
| **Diabetes** | E10-E14 | 5,245/3,049 | 14,628/10,341 | (2003-2007)/(2008-2011) |
| **COPD** | J44 | 1,445/1,310 | 3,970/2,991 | (2003-2008)/(2009-2011) |
| **Mental disorders** | F00-F99 | 3,021/1,743 | 14,471/3,320 | (2003-2009)/(2010-2011) |
| **Heart failure** | I50 | 2,173/2,029 | 5,567/4,570 | (2003-2008)/(2009-2011) |
| **Pneumonia** | J12-J18 | 24,52/2,828 | 6,170/6,439 | (2003-2008)/(2009-2011) |

Table 2: Predicting unplanned readmissions from the first day of each year after following the first diagnosis. using the Elixhauser comorbidities (Baselines) and automatically generated features (EMR only, and EMR with comorbidities). Prediction horizons of 1, 2, 3, 6, 12 months were considered. Confidence intervals of the AUCs were estimated using the Mann-Whitney method.

| **Disease** | **Prediction period** | **Baseline (1M)**  **(95% CI)** | **Baseline (3Y)**  **(95% CI)** | **Feature Set EMR**  **(95% CI)** | **Feature Set EMR+Comorbidities**  **(95% CI)** |
| --- | --- | --- | --- | --- | --- |
|  | 1M | 0.600 (0.557,0.642) | 0.652 (0.611,0.692) | **0.730 (0.695,0.766)** | 0.730 (0.695,0.766) |
|  | 2M | 0.613 (0.581,0.645) | 0.632 (0.600,0.665) | **0.719 (0.689,0.750)** | 0.719 (0.689,0.750) |
| **COPD** | , last three months | 0.615 (0.587,0.643) | 0.644 (0.616,0.672) | **0.719 (0.692,0.746)** | 0.720 (0.693,0.746) |
|  | 6M | 0.626 (0.603,0.649) | 0.627 (0.604,0.650) | **0.724 (0.703,0.746)** | 0.724 (0.702,0.745) |
|  | 12M | 0.632 (0.612,0.652) | 0.649 (0.629,0.669) | **0.720 (0.701,0.739)** | 0.720 (0.701,0.739) |
|  | 1M | 0.597 (0.560,0.634) | 0.639 (0.604,0.674) | **0.708 (0.674,0.741)** | 0.704 (0.670,0.738) |
|  | 2M | 0.622 (0.595,0.650) | 0.656 (0.629,0.682) | **0.718 (0.692,0.744)** | 0.718 (0.692,0.743) |
| **Diabetes** | , last three months | 0.629 (0.606,0.651) | 0.665 (0.643,0.686) | **0.724 (0.703,0.745)** | 0.724 (0.703,0.745) |
|  | 6M | 0.624 (0.607,0.642) | 0.657 (0.640,0.675) | **0.714 (0.697,0.731)** | 0.715 (0.698,0.732) |
|  | 12M | 0.641 (0.627,0.655) | 0.669 (0.655,0.683) | **0.718 (0.705,0.732)** | 0.718 (0.704,0.732) |
|  | 1M | 0.652 (0.609,0.695) | 0.624 (0.582,0.667) | **0.748 (0.709,0.787)** | 0.747 (0.708,0.786) |
|  | 2M | 0.660 (0.629,0.691) | 0.624 (0.592,0.657) | **0.756 (0.727,0.784)** | 0.756 (0.728,0.785) |
| **Mental disorders** | , last three months | 0.651 (0.624,0.679) | 0.617 (0.588,0.646) | **0.738 (0.713,0.764)** | 0.737 (0.711,0.762) |
|  | 6M | 0.630 (0.606,0.653) | 0.633 (0.609,0.656) | **0.718 (0.697,0.740)** | 0.718 (0.696,0.739) |
|  | 12M | 0.646 (0.625,0.667) | 0.637 (0.616,0.658) | **0.713 (0.694,0.732)** | 0.713 (0.694,0.732) |
|  | 1M | 0.665 (0.631,0.698) | 0.674 (0.641,0.707) | **0.749 (0.717,0.782)** | 0.750 (0.718,0.782) |
|  | 2M | 0.659 (0.633,0.685) | 0.692 (0.667,0.716) | **0.753 (0.729,0.777)** | 0.756 (0.733,0.780) |
| **Pneumonia** | , last three months | 0.657 (0.635,0.680) | 0.695 (0.674,0.716) | **0.760 (0.739,0.780)** | 0.762 (0.742,0.782) |
|  | 6M | 0.649 (0.631,0.667) | 0.687 (0.670,0.704) | **0.748 (0.731,0.764)** | 0.749 (0.733,0.765) |
|  | 12M | 0.661 (0.646,0.677) | 0.699 (0.685,0.714) | **0.744 (0.731,0.758)** | 0.747 (0.733,0.761) |

## Top features selected by the machine learning algorithm

From more than 8000 features generated from the administrative data, a machine learning algorithm with implicit feature selection ability was applied. To assess feature importance, we bootstrapped the data 100 times, and each bootstrap resulted in a subset of features being selected by the elastic net regularization Procedure. The importance of a feature is a product of its mean weight over all bootstraps and the standard deviation of the feature value over all derivation data units. These importance figures are then normalized so that the maximum absolute value is 100.

We also report here three other quantities: (i) Signal-to-Noise Ratio (SNR) is the ratio of the mean and standard deviation of the feature weight; (ii) mean weight, and (iii) selection probability is the chance of being selected by the regularization Procedure across all bootstraps.

The top 20 features selected the algorithm may help confirm known risk factors and reveal unknown ones. Features, whose selection probability is greater than 0.75, are ordered by their importance.

The following tables show the top features from two prediction horizons, the short-term (1 month) and the medium-term (12 months), for Diabetes and COPD:

- **Diabetes** (Table 3 , 1 month), (Table 4, 12 months),
- **COPD** (Table 5, 1 month), (Table 6, 12 months),
- **Mental disorders** (Table 7, 1 month), (Table 8, 12 months),
- **Pneumonia** (Table 9, 1 month), (Table 10, 12 months).

Table 3: Top features for Diabetes cohort, evaluated at unplanned discharges, 1 month prediction period. *Importance* is the product of feature mean weight and the standard deviation of the feature values over all training data points. *SNR* = Signal-To-Noise, the ratio between the mean weight of the feature and its standard deviation over 100 bootstraps. *Selec. prob.* is the probability that a feature is selected for each bootstrap.

| **Feature** | **Importance** | **SNR** | **Mean weight** | **Select. prob.** |
| --- | --- | --- | --- | --- |
| **Occupation: pensioner, retired & home duties** | 68.9 | 3.9 | 0.26 | 1.00 |
| **Length-of-stay, last month** | 31.9 | 1.9 | 0.26 | 0.97 |
| **Procedure code, last year: 1963 (Computerised tomography of abdomen and pelvis)** | 22.0 | 2.1 | 0.32 | 0.98 |
| **Diagnosis code, last month: Z86 (Personal history of certain other diseases)** | 16.3 | 1.8 | 0.27 | 0.97 |
| **Diagnosis code, last year: N77 (Vulvovaginal ulceration and inflammation in diseases classified elsewhere)** | 15.1 | 1.8 | 0.32 | 0.98 |
| **Procedure code, last year: 668 (Coronary angiography)** | 11.8 | 1.4 | 0.19 | 0.92 |
| **Diagnosis code, last month: R33 (Retention of urine)** | 11.7 | 2.8 | 0.58 | 1.00 |
| **Emergent admissions, last year** | 11.1 | 2.1 | 0.24 | 0.98 |
| **Emergent admissions, last year shift, last year** | 11.0 | 1.4 | 0.21 | 0.90 |
| **Diagnosis code, last six months: Z72 (Problems related to lifestyle)** | 10.9 | 1.8 | 0.21 | 0.95 |
| **DRG code count, last six months** | 10.4 | 3.1 | 0.28 | 1.00 |
| **Admissions, last six months** | 10.2 | 2.8 | 0.26 | 1.00 |
| **Length-of-stay, last six months** | 10.1 | 1.4 | 0.16 | 0.92 |
| **DRG code, the year before last year: F42A (Circulatory Disorders W/O AMI W Invasive Cardiac Inves Proc W Cat or Sev CC)** | 9.4 | 1.3 | 0.22 | 0.87 |
| **Age above 90** | 9.4 | 0.8 | 0.09 | 0.78 |
| **Diagnosis code, last three months Z72 (Problems related to lifestyle)** | 9.3 | 1.6 | 0.16 | 0.92 |
| **DRG code, last year: F72B (Unstable Angina W/O Catastrophic or Severe CC)** | 9.2 | 1.8 | 0.24 | 0.95 |
| **Admissions, last year** | 9.0 | 2.5 | 0.20 | 0.97 |
| **Diagnosis code, last six months: R51 (Headache)** | 8.6 | 2.0 | 0.26 | 0.96 |
| **Diagnosis code, the year before two years ago: I50 (Heart failure)** | 8.2 | 1.6 | 0.28 | 0.94 |

Table 4: Top features for Diabetes cohort, evaluated at unplanned discharges, 12 month prediction period. *Importance* is the product of feature mean weight and the standard deviation of the feature values over all training data points. *SNR* = Signal-To-Noise, the ratio between the mean weight of the feature and its standard deviation over 100 bootstraps. *Selec. prob.* is the probability that a feature is selected for each bootstrap.

| Feature | Importance | SNR | Mean weight | Select. prob. |
| --- | --- | --- | --- | --- |
| **Occupation: pensioner, retired & home duties** | 100.0 | 12.2 | 0.63 | 1.00 |
| **Emergent admissions, last year** | 34.7 | 5.3 | 1.23 | 1.00 |
| **Emergent admissions, year before last year** | 30.4 | 4.8 | 0.97 | 1.00 |
| **Admissions, last year** | 19.3 | 6.2 | 0.71 | 1.00 |
| **DRG counts, last year** | 16.5 | 5.5 | 0.72 | 1.00 |
| **Emergent admissions, year before two years ago** | 13.8 | 3.0 | 0.53 | 1.00 |
| **Procedure code: 1916 (Generalised allied health interventions)** | 11.8 | 1.6 | 0.25 | 0.94 |
| **Diagnosis code, last year: I20 (Angina pectoris)** | 11.3 | 2.5 | 0.39 | 0.99 |
| **Diagnosis code, last year** | 11.0 | 4.4 | 0.43 | 1.00 |
| **Diagnosis code, last month: Z86 (Personal history of certain other diseases)** | 10.9 | 2.2 | 0.30 | 0.97 |
| **Diagnosis code, the year before two years ago: I50 (Heart failure)** | 9.5 | 2.7 | 0.54 | 1.00 |
| **Length-of-stay, last three years** | 9.4 | 3.3 | 0.37 | 1.00 |
| **Age between 10-20** | 8.7 | 1.2 | 0.15 | 0.79 |
| **Procedure code, last year: 1963 (Computerised tomography of abdomen and pelvis)** | 8.5 | 1.3 | 0.20 | 0.88 |
| **Length-of-stay, last two years** | 8.2 | 3.2 | 0.33 | 1.00 |
| **DRG code, last year: F72B (Unstable Angina W/O Catastrophic or Severe CC)** | 7.8 | 2.2 | 0.34 | 0.99 |
| **DRG code, last year: F42A (Circulatory Disorders W/O AMI W Invasive Cardiac Inves Proc W Cat or Sev CC)** | 7.7 | 1.5 | 0.20 | 0.91 |
| **Diagnosis code, last year: Z72 (Problems related to lifestyle)** | 7.7 | 2.2 | 0.29 | 0.98 |
| **Diagnosis code, the year before last year: J98 (Other respiratory disorders)** | 7.7 | 1.4 | 0.25 | 0.94 |
| **Length-of-stay, last month** | 7.6 | 0.9 | 0.10 | 0.77 |

Table 5: Top features for COPD cohort, evaluated at unplanned discharges, 1 month prediction period. *Importance* is the product of feature mean weight and the standard deviation of the feature values over all training data points. *SNR* = Signal-To-Noise, the ratio between the mean weight of the feature and its standard deviation over 100 bootstraps. *Selec. prob.* is the probability that a feature is selected for each bootstrap.

| **Feature** | **Importance** | **SNR** | **Mean weight** | **Select. prob.** |
| --- | --- | --- | --- | --- |
| **Emergent admissions last year** | 38.9 | 3.5 | 0.53 | 1.00 |
| **Occupation: pen, retired & home duties** | 33.0 | 1.5 | 0.14 | 0.92 |
| **Male** | 31.1 | 1.4 | 0.09 | 0.91 |
| **Diagnosis code, the year before two years ago: I50 (Heart failure)** | 22.0 | 1.7 | 0.33 | 0.96 |
| **Diagnosis code, last month: I50 (Heart failure)** | 20.0 | 1.4 | 0.21 | 0.88 |
| **Diagnosis code, last year: T89 (Other specified complications of trauma)** | 19.2 | 1.7 | 0.32 | 0.92 |
| **Procedure, last three months: 2006 (Lung perfusion or ventilation study)** | 17.6 | 1.3 | 0.16 | 0.87 |
| **Emergent admissions, the year before last year** | 17.1 | 1.3 | 0.21 | 0.88 |
| **DRG code, last year: E65B (Chronic Obstructive Airways Disease W/O Catastrophic CC)** | 16.9 | 1.5 | 0.21 | 0.92 |
| **DRG code, last six months: E65B (Chronic Obstructive Airways Disease W/O Catastrophic CC)** | 16.6 | 1.7 | 0.21 | 0.93 |
| **Procedure, last three months: 544 (Bronchoscopy with biopsy or removal of foreign body)** | 15.0 | 1.3 | 0.18 | 0.88 |
| **Diagnosis code, last month: Z86 (Personal history of certain other diseases)** | 13.4 | 1.1 | 0.13 | 0.84 |
| **Procedure, last month: 544 (Bronchoscopy with biopsy or removal of foreign body)** | 13.3 | 1.5 | 0.23 | 0.89 |
| **Diagnosis code last year: R45 (Symptoms and signs involving emotional state)** | 13.3 | 1.2 | 0.21 | 0.83 |
| **Diagnosis code, the year before two years ago: J44 (Other chronic obstructive pulmonary disease)** | 12.4 | 1.5 | 0.20 | 0.91 |
| **Diagnosis code, last year: J22 (Unspecified acute lower respiratory infection)** | 12.3 | 1.1 | 0.14 | 0.77 |
| **Diagnosis code, last year: E87 (Other disorders of fluid, electrolyte and acid-base balance)** | 12.3 | 1.1 | 0.17 | 0.83 |
| **Diagnosis code, last year: Y51 (Drugs primarily affecting the autonomic nervous system causing adverse effects in therapeutic use)** | 11.1 | 1.2 | 0.18 | 0.81 |
| **Diagnosis code, last year: J96 (Respiratory failure, not elsewhere classified)** | 10.0 | 1.2 | 0.17 | 0.79 |
| **Admissions, last six months** | 9.6 | 1.3 | 0.13 | 0.83 |

Table 6: Top features for COPD cohort, evaluated at unplanned discharges, 12 month prediction period. *Importance* is the product of feature mean weight and the standard deviation of the feature values over all training data points. *SNR* = Signal-To-Noise, the ratio between the mean weight of the feature and its standard deviation over 100 bootstraps. *Selec. prob.* is the probability that a feature is selected for each bootstrap.

| **Feature** | **Importance** | **SNR** | **Mean weight** | **Select. prob.** |
| --- | --- | --- | --- | --- |
| **Occupation: pen, retired & home duties** | 100.0 | 7.6 | 0.68 | 1.00 |
| **Emergent admissions, last year** | 48.3 | 5.4 | 1.07 | 1.00 |
| **Emergent admissions, year before last year** | 36.1 | 4.3 | 0.73 | 1.00 |
| **Procedure last year: 1916 (Generalised allied health interventions)** | 35.3 | 3.9 | 0.61 | 1.00 |
| **DRG code last year: E65B (Chronic Obstructive Airways Disease W/O Catastrophic CC)** | 30.7 | 3.9 | 0.61 | 1.00 |
| **DRG counts, last six months** | 12.9 | 2.4 | 0.30 | 1.00 |
| **DRG count, last year** | 12.4 | 4.0 | 0.41 | 1.00 |
| **Male** | 11.9 | 1.1 | 0.06 | 0.86 |
| **Diagnosis code last year: R07 (Pain in throat and chest)** | 10.2 | 1.8 | 0.31 | 0.98 |
| **Diagnosis code last year** | 9.4 | 2.5 | 0.25 | 0.98 |
| **DRG code last year: C08Z** | 9.2 | 1.2 | 0.21 | 0.88 |
| **Admissions last year** | 8.1 | 2.8 | 0.28 | 1.00 |
| **Diagnosis code last year: R45 (Symptoms and signs involving emotional state)** | 7.9 | 1.2 | 0.21 | 0.85 |
| **DRG counts, the year before two years ago** | 7.5 | 2.1 | 0.24 | 0.97 |
| **Diagnosis code last year: J96 (Respiratory failure, not elsewhere classified)** | 7.2 | 1.2 | 0.19 | 0.85 |
| **Diagnosis code, the year before two years ago: Z53 (Persons encountering health services for specific Procedures, not carried out)** | 6.8 | 1.2 | 0.20 | 0.85 |
| **Diagnosis code last year: J44 (Other chronic obstructive pulmonary disease)** | 6.8 | 1.0 | 0.13 | 0.75 |
| **DRG code, the year before last year: G67B (Oesophagitis and Gastroenteritis W/O Cat/Sev CC)** | 6.5 | 1.4 | 0.25 | 0.91 |
| **Diagnosis code, the year before two years ago: J44 (Other chronic obstructive pulmonary disease)** | 6.4 | 1.1 | 0.17 | 0.80 |
| **Diagnosis code, the year before two years ago: R07 (Pain in throat and chest)** | 6.2 | 1.2 | 0.20 | 0.83 |

Table 7: Top features for Mental disorders cohort, evaluated at unplanned discharges, 1 month prediction period. *Importance* is the product of feature mean weight and the standard deviation of the feature values over all training data points. *SNR* = Signal-To-Noise, the ratio between the mean weight of the feature and its standard deviation over 100 bootstraps. *Selec. prob.* is the probability that a feature is selected for each bootstrap.

| **Feature** | **Importance** | **SNR** | **Mean weight** | **Select. prob.** |
| --- | --- | --- | --- | --- |
| **Emergent admissions, last year** | 91.3 | 3.3 | 0.58 | 1.00 |
| **Occupation: pen, retired & home duties** | 91.1 | 2.2 | 0.11 | 0.98 |
| **Procedure, last month: 668 (Coronary angiography)** | 47.1 | 1.9 | 0.31 | 0.99 |
| **Length-of-stay, last month** | 43.5 | 1.2 | 0.15 | 0.81 |
| **Diagnosis code, last year: R10 (Abdominal and pelvic pain)** | 40.6 | 2.4 | 0.37 | 1.00 |
| **Procedure, last month: 1893 (Administration of blood and blood products)** | 34.6 | 2.4 | 0.45 | 1.00 |
| **Procedure, the year before two years ago: 1909 (Conduction anaesthesia)** | 34.2 | 1.6 | 0.29 | 0.90 |
| **Diagnosis code, last six months: X59 (Exposure to unspecified factor)** | 33.0 | 2.0 | 0.30 | 0.98 |
| **DRG counts, last six months** | 32.9 | 2.1 | 0.23 | 0.99 |
| **Emergent admissions, last three months** | 30.7 | 2.0 | 0.21 | 0.97 |
| **Emergent admissions, last six months** | 30.7 | 2.0 | 0.21 | 0.97 |
| **Diagnosis code, last six months: F60 (Specific personality disorders)** | 30.5 | 2.3 | 0.27 | 0.98 |
| **Diagnosis code, last year: K29 (Gastritis and duodenitis)** | 30.3 | 1.6 | 0.28 | 0.96 |
| **Admissions, last six months** | 29.7 | 2.2 | 0.21 | 0.99 |
| **Rare-diagnosis code, last six months** | 26.0 | 1.2 | 0.16 | 0.84 |
| **Age between 10-20** | 26.0 | 0.9 | 0.06 | 0.75 |
| **ED code, last three months: I21 (Acute myocardial infarction)** | 24.6 | 1.5 | 0.20 | 0.89 |
| **Diagnosis code, last six months: Y92 (Place of occurrence)** | 24.4 | 1.6 | 0.21 | 0.93 |
| **DRG code, last year: X62B (Poisoning/Toxic Effects of Drugs and Other Substances W/O Cat or Sev CC)** | 24.3 | 1.5 | 0.22 | 0.91 |
| **Emergent admissions, last month** | 22.2 | 1.5 | 0.17 | 0.95 |

Table 8: Top features for Mental disorders cohort, evaluated at unplanned discharges, 12 month prediction period. *Importance* is the product of feature mean weight and the standard deviation of the feature values over all training data points. *SNR* = Signal-To-Noise, the ratio between the mean weight of the feature and its standard deviation over 100 bootstraps. *Selec. prob.* is the probability that a feature is selected for each bootstrap.

| **Feature** | **Importance** | **SNR** | **Mean weight** | **Select. prob.** |
| --- | --- | --- | --- | --- |
| **Occupation: pen, retired & home duties** | 100.0 | 10.3 | 0.50 | 1.00 |
| **Emergent admissions, last year** | 55.2 | 5.6 | 1.39 | 1.00 |
| **Diagnosis code, last year** | 28.0 | 5.2 | 0.70 | 1.00 |
| **Emergent admissions, the year before last year** | 19.8 | 3.4 | 0.56 | 1.00 |
| **DRG code count, last year** | 16.9 | 5.4 | 0.61 | 1.00 |
| **Admissions, last year** | 13.8 | 4.9 | 0.50 | 1.00 |
| **Age between 10-20** | 13.8 | 1.6 | 0.13 | 0.94 |
| **Diagnosis code, the year before last year: Z72 (Problems related to lifestyle)** | 13.1 | 2.1 | 0.40 | 1.00 |
| **Emergent admissions, the year before two years ago** | 12.5 | 2.0 | 0.38 | 0.98 |
| **DRG code counts, last six months** | 8.6 | 2.0 | 0.24 | 0.94 |
| **Diagnosis code, last year: Z72 (Problems related to lifestyle)** | 8.5 | 1.9 | 0.28 | 0.97 |
| **Diagnosis code, last six months: R07 (Pain in throat and chest)** | 7.8 | 1.9 | 0.25 | 0.99 |
| **Procedure, the year before two years ago: 1916 (Generalised allied health interventions)** | 7.6 | 1.6 | 0.31 | 0.97 |
| **Emergency attendance time, last year** | 7.4 | 1.6 | 0.15 | 0.93 |
| **Emergency attendance time, last six months** | 7.4 | 1.5 | 0.18 | 0.94 |
| **Diagnosis code, last six months: F60 (Specific personality disorders)** | 7.4 | 2.1 | 0.26 | 1.00 |
| **Diagnosis code, last year: J44 (Other chronic obstructive pulmonary disease)** | 7.3 | 2.3 | 0.28 | 0.99 |
| **Diagnosis code, last year: G43 (Migraine)** | 7.0 | 1.8 | 0.32 | 0.96 |
| **Diagnosis code, last six months** | 6.9 | 2.3 | 0.22 | 0.99 |
| **Diagnosis code, last year: R45 (Symptoms and signs involving emotional state)** | 6.9 | 1.4 | 0.19 | 0.91 |

Table 9: Top features for Pneumonia cohort, evaluated at unplanned discharges, 1 month prediction period. *Importance* is the product of feature mean weight and the standard deviation of the feature values over all training data points. *SNR* = Signal-To-Noise, the ratio between the mean weight of the feature and its standard deviation over 100 bootstraps. *Selec. prob.* is the probability that a feature is selected for each bootstrap.

| **Feature** | **Importance** | **SNR** | **Mean weight** | **Select. prob.** |
| --- | --- | --- | --- | --- |
| **Occupation: pen, retired & home duties** | 57.7 | 2.0 | 0.19 | 0.97 |
| **Male** | 46.7 | 2.0 | 0.14 | 0.98 |
| **Emergent admissions, last year** | 44.8 | 2.9 | 0.54 | 1.00 |
| **Emergent admissions, the year before last year** | 20.3 | 1.8 | 0.27 | 0.93 |
| **Emergent admissions, last three months** | 19.8 | 2.2 | 0.26 | 0.99 |
| **Emergent admissions, last six months** | 19.8 | 2.2 | 0.26 | 0.99 |
| **Diagnosis code, the year before two years ago: E86 (Volume depletion)** | 18.6 | 1.8 | 0.37 | 0.97 |
| **Diagnosis code, last month: J44 (Other chronic obstructive pulmonary disease)** | 17.7 | 1.4 | 0.18 | 0.90 |
| **Age between 20-30** | 17.3 | 1.0 | 0.15 | 0.75 |
| **Procedure code, last month: 1916 (Generalised allied health interventions)** | 17.3 | 1.3 | 0.13 | 0.84 |
| **Diagnosis code, last year: Y92 (Place of occurrence)** | 15.2 | 1.4 | 0.19 | 0.87 |
| **Procedure, the year before two years ago: 1916 (Generalised allied health interventions)** | 13.6 | 1.6 | 0.25 | 0.96 |
| **Diagnosis code, last year: J18 (Pneumonia, organism unspecified)** | 13.5 | 1.2 | 0.16 | 0.87 |
| **Procedure counts, last month** | 12.3 | 1.4 | 0.17 | 0.86 |
| **DRG code, the year before last year: E62C (Respiratory Infections/Inflammations W/O CC)** | 11.9 | 1.3 | 0.22 | 0.89 |
| **DRG code, last year: F60A (Circulatory Disorders W AMI W/O Invasive Cardiac Inves Proc W Catastrophic CC)** | 11.7 | 1.6 | 0.23 | 0.94 |
| **Diagnosis code, last year: B37 (Candidiasis)** | 11.4 | 1.4 | 0.24 | 0.90 |
| **Diagnosis code, the year before two years ago: I50 (Heart failure)** | 11.2 | 1.6 | 0.26 | 0.88 |
| **Procedure, last year: 1963 (Computerised tomography of abdomen and pelvis)** | 11.1 | 1.8 | 0.24 | 0.99 |
| **Diagnosis code, the year before last year: R79 (Other abnormal findings of blood chemistry)** | 10.8 | 1.3 | 0.25 | 0.90 |

Table 10: Top features for Pneumonia cohort, evaluated at unplanned discharges, 12 month prediction period. *Importance* is the product of feature mean weight and the standard deviation of the feature values over all training data points. *SNR* = Signal-To-Noise, the ratio between the mean weight of the feature and its standard deviation over 100 bootstraps. *Selec. prob.* is the probability that a feature is selected for each bootstrap.

| **Feature** | **Importance** | **SNR** | **Mean weight** | **Select. prob.** |
| --- | --- | --- | --- | --- |
| **Occupation: pen, retired & home duties** | 100.0 | 8.0 | 0.75 | 1.00 |
| **Emergent admissions, last year** | 49.4 | 4.6 | 1.33 | 1.00 |
| **Male** | 30.9 | 3.2 | 0.21 | 1.00 |
| **Emergent admissions, the year before last year** | 20.2 | 3.3 | 0.60 | 1.00 |
| **Procedure, last year: 1916 (Generalised allied health interventions)** | 19.4 | 3.0 | 0.50 | 1.00 |
| **Age between 80-90** | 11.2 | 1.3 | 0.08 | 0.92 |
| **Diagnosis code counts, last year** | 10.0 | 3.2 | 0.33 | 1.00 |
| **Diagnosis code, the year before last year: P92 (Feeding problems of newborn)** | 9.4 | 2.4 | 0.50 | 0.99 |
| **DRG code counts, last year** | 9.1 | 3.5 | 0.39 | 1.00 |
| **Emergent admissions, the year before two years ago** | 8.3 | 1.8 | 0.30 | 0.94 |
| **Admissions, the year before two years ago** | 7.5 | 3.5 | 0.34 | 1.00 |
| **Diagnosis code counts, last six months** | 6.5 | 1.9 | 0.22 | 0.98 |
| **Diagnosis code, last year: J44 (Other chronic obstructive pulmonary disease)** | 6.5 | 1.4 | 0.21 | 0.88 |
| **Diagnosis code, last year: B34 (Viral infection of unspecified site)** | 6.1 | 1.9 | 0.33 | 0.96 |
| **Diagnosis code, last month: J44 (Other chronic obstructive pulmonary disease)** | 6.0 | 1.0 | 0.14 | 0.81 |
| **DRG code counts, the year before two years ago** | 5.9 | 2.6 | 0.25 | 1.00 |
| **DRG code, last three months: F60A (Circulatory Disorders W AMI W/O Invasive Cardiac Inves Proc W Catastrophic CC)** | 5.8 | 1.8 | 0.24 | 0.96 |
| **Diagnosis code, last year: I10 (Essential (primary) hypertension)** | 5.7 | 1.5 | 0.20 | 0.94 |
| **Procedure, last six months: 1916 (Generalised allied health interventions)** | 5.2 | 1.1 | 0.12 | 0.76 |
| **Diagnosis code, last month: Z72 (Problems related to lifestyle)** | 5.2 | 1.6 | 0.25 | 0.95 |
